# Childhood adversity and psychopathology: the dimensions of timing, type and chronicity in a population-based sample of high-risk adolescents

**DOI:** 10.1186/s13034-024-00727-x

**Published:** 2024-03-18

**Authors:** Milan Zarchev, Nina H. Grootendorst-van Mil, Diandra C. Bouter, Witte J. G. Hoogendijk, Cornelis L. Mulder, Astrid M. Kamperman

**Affiliations:** 1 Department of Psychiatry, Erasmus University Medical Center, P.O. box 2040, 3000 CA Rotterdam, the Netherlands; 2 Epidemiological and Social Psychiatric Research Institute (ESPRi), Department of Psychiatry, Erasmus University Medical Center, P.O. box 2040, 3000 CA Rotterdam, the Netherlands; 3 Antes Mental Health Care, Parnassia Psychiatric Institute, Albrandswaardsedijk 74, 3172 AA Poortugaal, the Netherlands

**Keywords:** Adversity, Psychopathology, Early life stress

## Abstract

**Background:**

Research on childhood adversity and psychopathology has begun investigating the dimension of timing, however the results have been contradictory depending on the study population, outcome and how adverse life events (ALEs) were operationalized. Additionally, studies so far typically focus only on a narrow range of psychiatric diagnoses or symptoms. The current cross-sectional study aimed to examine the association between timing, type and chronicity of ALEs and adolescent mental health problems.

**Methods:**

Adolescents from a population-based cohort oversampled on emotional and behavioral problems (mean age 14.8; range 12–17, N = 861) were included in the current analysis. Primary caregivers were interviewed on what ALEs adolescents experienced. ALEs were defined in two ways: (1) broad operationalization, including school difficulties, parental divorce, and family sickness; and (2) physically threatening abuse only, including physical and sexual violence. After looking at lifetime ALEs, we turned to chronicity, timing and sex differences. We focused on overall psychiatric symptoms as well as specific domains of emotional and behavioral problems, assessed using the Youth Self Report (YSR) and psychotic experiences assessed using the Prodromal Questionnaire-16 (PQ-16). A series of linear models adjusted for sociodemographic and parental factors were used.

**Results:**

Lifetime ALEs were associated with all types of psychopathology, with relatively bigger effect sizes for broad than for physical ALEs. The latter associations were found to be more robust to unmeasured confounding. The 9–12 age period of experiencing both broad and physical ALE’s was most saliently associated with any psychopathology. Girls were more at risk after experiencing any ALEs, especially if the adversity was chronic or ALEs took place after the age of 12.

**Conclusions:**

Broad as well as physical ALEs are associated with psychopathology, especially ALEs experienced during the 9–12 age period. Physical ALEs may be more useful in investigating specific etiological factors than broad ALEs. Sex differences may not emerge in lifetime measures of ALEs, but can be important for chronic and later childhood adversity.

**Supplementary Information:**

The online version contains supplementary material available at 10.1186/s13034-024-00727-x.

## Introduction

Adverse life experiences (ALEs) are some of the most robust environmental predictors of future psychological, social and economic outcomes [31, 73]. The original Adverse Childhood Experiences (ACE) study performed by the Centers for Disease Control and Prevention in the mid-1990s demonstrated a marked dose–response relationship between the count of childhood adversities and increased problems in the affective, cognitive, somatic, aggression and substance use domains [3]. This operationalization of counting the number of specific adversities was named the ACE score and it attracted substantial interest as both a tool for screening and for studying etiological factors of disease [30, 60]. The use of the cumulative ACE score, however, has been challenged on both these fronts [26, 55]. A person who experienced parental incarceration at age four could conceivably receive the same ACE score as a teenager who was subjected to sexual abuse at fifteen. This has produced criticisms that the current approach neglects critical dimensions of adversity, such as the type (e.g., parental deprivation versus threatening abuse) and timing (e.g., age, occurrence and frequency) of the adverse events. These aspects can significantly influence the impact of childhood experiences, yet are overlooked in the cumulative scoring method [4, 54]. Attention has therefore shifted to investigating how type and timing of adversity can account for the development of emotional and behavioral problems.

Extensive research has been devoted to investigating the association between specific types of abuse and psychopathology [10, 32, 45]. Isolating distinct adversity categories, however, is not straightforward as co-occurrence of multiple childhood events is overwhelmingly the norm rather than the exception [14]. For instance, up to 63% of people that report one type of childhood maltreatment also report other abuse [80] Thus, rather than replacing the original ACE list with individual adverse events, researchers have proposed several aspects on which cumulative approaches can be improved. Two will be highlighted here. First, a more comprehensive set of adversities guided by previous research has been suggested to expand the list of adversities [27]. Less visible events such as peer and school difficulties, constant moving and a disorganized family environment all pose unique developmental challenges that can be incorporated in a broader operationalization of adversity [25, 70, 75]. Authors have advocated for using different terminology to differentiate this broader operationalization from the original ACE score, like for example early adversity or adverse life events (ALEs) [12]. Second, while individual events might be impossible to isolate, salient characteristics shared by groups of events can be investigated separately. One theoretical research line considers threatening events (such as physical or sexual abuse) separately from other adverse experiences which might be more depriving in nature (such as losing a parent, neglect, academic problems) [54]. Empirical investigations using the latter approach are still in early stages and studies are yet to compare using a broad cumulative operationalization of ALEs including all events compared to ALEs consisting exclusively of physically threatening events such as sexual and physical abuse. Exploratory studies using these new operationalizations are needed to investigate the development of emotional, behavioral and cognitive problems similar to the tradition of the original ACE methodology.

Beside the issue of grouping events, the dimension of timing is also key in understanding the mechanisms through which adversity operates. According to theory, as the developing brain matures, different brain structures undergo periods of heightened neuroplasticity and accordingly periods of heightened vulnerability to stress as well [47]. For example, the hippocampus roughly develops in the first 5 years of life, after which the amygdala goes through a period of increased sensitivity in the ages of 8 to 12, while the prefrontal cortex peaks later in the teenage years [46]. Building on that knowledge, researchers started exploring the distal effects of stress in relation to how its timing affects psychopathological outcomes. The empirical findings have been mixed. Some researchers report that physical and sexual abuse in childhood were associated with depressive problems, anxiety symptoms, and suicidal ideation regardless of when they happened, as found in both large cohorts [20, 22] and longitudinal designs [18]. Other studies report adversity before adolescence (e.g. before age 11) to be more predictive of psychosis and depression when compared to adversity in adolescence [2, 50, 66]. Yet others report adversity before age 6 as most predictive of anxiety and depression problems [43]. Finally, timing of adversity has sometimes been reported not to be of consequence to transitioning into psychiatric disorders, as only cumulative lifetime scores were found to be significantly associated with psychopathology [20, 22, 35]. These findings are difficult to compare and combine due to several design differences, which can be found in the sample populations; the outcomes under study and the analysis strategies used. To form a solid knowledge base, studies investigating a broad range of outcomes covering the internalizing/externalizing spectrum are necessary. This approach is analogous to the outcome-wide epidemiologic approach [78]. Briefly, the outcome-wide approach argues that it is desirable to relate a single category of exposure to many health outcomes in a single study while controlling for shared sources of bias. This approach has rarely been applied in the context of childhood adversity. In particular the timing dimension of adversity has not been studied in relation to a variety of mental health outcomes using a single sample and analysis strategy.

Finally, childhood adversities affect men and women differently. Women are 1.5 to 5 times more likely to develop anxious or depressive responses to adverse events [37, 69]. Beyond emotional problems, women are also more likely to report behavioral problems following childhood adversity [24]. These differences are not present in early childhood, but emerge around ages 11 to 13 when girls also start developing depressive problems at an exponentially higher rate [7, 63]. While biological differences in this early pubertal period have been a leading hypothesis for the mechanism behind observed sex differences, interactions between (neuro)hormonal factors and adversity can only account for up to a quarter of the variance in psychopathology [13, 67]. Wider social factors clearly have an important role in this relationship, some of which could be captured by broader inclusion of adverse events. Regardless of how social or biological the etiology behind observed differences is, it is clear that sex is an important moderator when studying the relationship between adversity and psychopathology.

The current cross-sectional study investigates the dimensions of type and timing of ALEs and their association with psychopathology in a population-based cohort of adolescents. We take an outcome-wide approach to examine a wide range of emotional and behavioral psychopathology domains as outcomes. Those include anxious-depressed, withdrawn-depressed, somatic, rule-breaking, aggressive, social, attention, and thought problems, as well as psychotic experiences. To investigate the dimension of type of adversity, we scored ALEs in two ways: one which includes a wide array of adverse experiences (broad operationalization) and one which separately considers physically threatening experiences (e.g. sexual and physical violence) in line with distinctions made in the theoretical literature. To investigate timing, threatening and broad adverse life events were categorized according to when they occurred, namely in the first 3 years of life, between ages 4 to 8, ages 9 to 12, or after the age of 12. While we hypothesize a positive association between adversity and psychopathology, past literature is equivocal on specifying which periods of life should be particularly vulnerable to adversity. We therefore do not pre-specify which period we expect to be most associated with psychopathology. Third, we address the role of sex in moderating the association with psychopathology.

## Methods

### Setting and study population

The current study uses the iBerry (Investigating Behavioral and Emotional Risk in Rotterdam Youth) cohort, which follows 1022 adolescents [34]. The sampling method was designed to deliberately include more adolescents at risk of developing emotional and behavior problems and thus increase the statistical power of analyses targeting mental health outcomes [52]. The selection process started with 16,758 adolescents in their first year of high school (aged 13), who were screened using the self-report Strengths and Difficulties Questionnaire (SDQ [33]). All the adolescents who scored in the highest 15% range of the SDQ problem scores and a random sample of the bottom 85% were included, resulting in a 2.5:1 ratio of high-risk adolescents. For the current cross-sectional analysis, adversity and psychopathology data were available for 861 (84.2%) out of the 1022 adolescents during the baseline assessment (mean age 14.8 years).

### Adverse life events predictors

Information on exposure to adverse life events during childhood was obtained via the childhood adversity interview from The Tracking Adolescents' Individual Lives Survey [58]. The accompanying parent or caregiver was asked whether each of 13 events occurred in the adolescent’s life and how old the adolescent was at the time of the event. These events were: (1) hospitalization of the adolescent, (2) serious illness or hospitalization of the mother, (3) father, (4) sibling or (5) close friend; (6) death in the family or (7) outside of family (e.g. of a friend); (8) parental divorce; (9) repeating class; (10) switching school; (11) extended living away from home; (12) physical violence and (13) sexual abuse. We categorized whether each event happened in the following four age periods: 0 to 3 years old; 4 to 8; 9 to 12; 12 or later. Chronicity was measured as in how many of these four age periods an event had occurred. These intervals were chosen to balance theoretically meaningful maturation windows which are considered particularly susceptible to the effects of adverse life events [5] and to also keep in line with adverse life event timing literature so far [19]. A cumulative sum score theoretically ranging between 0 (no events experienced) and 13 (all events experienced) was created. This all-inclusive broad score, which includes physical and sexual abuse, is closest to the cumulative scores used in prior literature [72]. Additionally, cumulative sum scores for each of the four age periods were created (e.g. 4 events that occurred in the 4 to 8 years old period). Certain events were not included in the 0 to 3 year olds period, as they were impossible to occur (repeating class and switching school). Finally, the same sum scores were applied for only physical and sexual abuse events to create a sum score of physically threatening adverse events ranging from 0 to 2 (both physical and sexual abuse occurred). This latter sum score was also split into timing periods, however this time each variable was a binary indicator if sexual and/or physical abuse had occurred. These events were isolated as physical and particularly traumatizing, as they are interpersonal, assaultive and generally considered within the psychiatric literature as particularly traumagenic [9].

### Psychopathology outcomes

Two self-report questionnaires were used to collect data on the adolescents’ emotional and behavioral problems. The Youth Self-Report scale (YSR; [1]) was used to measure general internalizing and externalizing problems, as well as a composite total psychological problems score. Problem subscales were also calculated, namely for anxious-depressive problems, withdrawn-depressive problems, somatic complaints, social problems, thought problems, attention problems, rule-breaking behavior and aggressive behavior. The questionnaire is comprised of 112 items, which could be answered on a 3-point Likert scale (“Not true”,“Somewhat or sometimes true”; “Always true”). Detailed information on number of items for each subscale and reliability estimates is presented in Additional file 1: Table S1. McDonald’s omega ranged from 0.95 for total problems to 0.55 for social problems. The 16-item version of the Prodromal Questionnaire (PQ-16) was used as a measurement of adolescents’ psychotic experiences [38]. This questionnaire asks about hallucinatory experiences (e.g. “I see things others don’t”), delusional ideations (e.g. “I sometimes find special meaning in advertisements”) and two items on negative symptoms (e.g. “I don’t find interest in things I used to enjoy”) associated with risk for developing a psychotic disorder. The response options for each item are a binary “Agree” or “Disagree”. The items are summed together in a total score, with good reliability–McDonald’s omega was 0.68 for boys and 0.73 for girls.

### Confounder variables

Basic demographic information about age and sex was collected from the participating adolescents using self-report questionnaires. Educational achievement was categorized according to the Dutch high school system. The lowest level was pre-vocational secondary education, followed by higher-general secondary education, then pre-university general education. There were also two categories for combined education levels and special needs education. The accompanying caregiver (primarily mothers, 83.3%) provided information on household income (categorized as≤ € 1599, € 1600–2399, € 2400–4399,≥ € 4400). Ethnic origin was categorized as one of the parents being born abroad (e.g. Dutch vs. non-Dutch). Urbanicity of living environment was determined from the number of addresses per km^2^ surrounding the adolescents’ home address; each adolescent was categorized as living rural, suburban or urban neighborhood (defined as< 1000, 1000–1500, and> 1500 addresses/km^2^, respectively)[71]. Parental psychopathology was assessed using the Brief Symptoms Inventory (BSI [15]). The BSI is a self-report general measure consisting of 53 items with 3-point Likert-scale response options each, measuring psychological symptoms in various domains such as depression, anxiety, somatization, hostility, psychoticism and paranoid ideation. A weighted mean global severity index measuring the general psychological functioning was calculated.

### Statistical analysis

The estimates of interest in the current analysis were the associations of adverse life events as predictors and a range of emotional and behavioral problems as outcomes. The broad operationalization of ALE produced the following set of predictors: (1) a lifetime cumulative score; (2) broad ALEs occurring before age 3; (3) occurring between ages 4 to 8; (4) occurring between ages 8 to 12; (5) occurring after age 12; (6) chronicity of broad ALEs. The physically threatening operationalization produced identical six predictors, however in this case only sexual and/or physical abuse was counted as an ALE. Each broad and physical ALE predictor was modelled separately. For the outcomes, the following 12 problems were considered: (1) total psychopathological problems; (2) externalizing problems; (3) internalizing problems; (4) anxious-depressive problems; (5) withdrawn-depressive problems; (6) somatic complaints: (7) social problems; (8) thought problems; (9) attention problems; (10) rule-breaking behavior; (11) aggressive behavior and (12) psychotic experiences. We underscore that our approach to this analysis is primarily exploratory. Individually there are 12 predictors times 12 outcomes resulting in 144 associations. Consequently, individual p-values are not adjusted, nor interpreted in isolation as independent tests, as this would require more statistical power than any existing individual cohort can offer. Instead they are used to identify patterns on a general level in timing, type of ALE and psychopathology. Each of the specific 144 associations could then be the subject of careful confirmatory planning in a future study.

For the current cross-sectional analyses we used multiple linear regression models. Each model was adjusted for a set of demographic and familial confounders. These were age, sex, ethnic origin, educational level, urbanicity of living environment, parental psychopathology and harsh parenting from the primary caregiver. The association between later-life ALEs and psychopathology could be confounded by a history of chronic ALEs earlier in life. This is why to adjust for earlier ALEs, the association with the 9–12 and 12 and older timing predictors, we also included the chronicity variable as a confounder. As an estimate of the effect size between the ALE predictors and a given outcome, Pearson’s semi-partial r was used. This allowed us to plot all associations and compare them in a standardized way. The significance level was set at the standard 0.05 for exploratory research [28]. As a secondary analysis, we also added interaction terms between the ALE predictor and sex to each model to test which associations were moderated by sex. We present standardized interaction coefficients from these analyses to compare the direction and magnitude of moderation for each psychopathology outcome. A series of effect plots are presented to assess how associations differ between sexes. All regression analyses were performed using the base R statistical software (v. 4.1.1 [61]). Additionally, we calculated estimates for unmeasured confounders, which would have to be present to completely explain the observed associations. Briefly, the procedure as implemented in the R packages “sensemakr” calculates how many times an unobserved confounder would have to be bigger than an observed one for the association of interest to become zero [11]. We selected parental psychopathology as a large and pervasive confounder against which to benchmark potential unobserved confounders.

Because some adolescents participated without one of their parents or caregiver, there were 112 (10.9%) participants with missing data for the adverse life events variables. Those cases were not included, as there were no adequate auxiliary variables to carry out an imputation with. Missing case analysis is presented in Additional file 1: Table S2. It showed that compared to those who contributed ALE data, the missing 112 participants score higher on rule-breaking behavior (p = 0.002). There were a further 49 participants with missing psychopathology data, leaving the analysis sample with 861 participants. In this final analysis sample, there were 58 participants missing data for household income, 45 for harsh parenting, 44 for parental psychopathology, 31 for ethnic origin and 9 for education. Multiple imputation as implemented in the R package “mice” was used to replace these covariate missing values with 5 imputed datasets [77]. Auxiliary variables used only for imputation were IQ scores [74], perceived social support [81] and sense of coherence [6].

All analysis files are available at https://osf.io/8w7bp/.

## Results

Characteristics of the included adolescents stratified by sex are presented in Table 1. The analysis sample comprised 861 adolescents with mean age of 14.9 years (ranging from 12.6 to 18.1) and 48% were male. The majority of participants were of Dutch origin (78%) and lived in an urban setting (60%). On average, boys reported similar rates of adversities (mean 5.85 lifetime events) as girls (mean 5.86 lifetime events). Of note, only 15 adolescents had physical or sexual abuse reported before age 3. This limits considerably the inferential evidence that can be obtained for associations in this combination of age group and type of ALE. The full distribution of ALEs across age groups is presented in Additional file 1: Table S3. Girls had a higher average score on total psychological problems (46) than boys (39, see Additional file 1: Table S4). Across the sample, the mean Pearson’s correlation between psychopathology outcomes was r = 0.54 (SD = 0.18).

**Table 1:** Characteristics of the adolescents (N = 861)

	Male n = 414^*a*^	Female n = 447^*a*^
Age, years	14.87 (14.35, 15.30)	14.84 (14.33, 15.26)
Ethnic origin		
Dutch	308 (77%)	338 (78%)
Non-Dutch	90 (23%)	94 (22%)
Net monthly household income, euro’s		
< 1599€	47 (12%)	51 (12%)
1600–2399€	57 (15%)	72 (17%)
2400–4399€	192 (50%)	209 (50%)
> 4400€	91 (23%)	84 (21%)
Educational level		
Pre-vocational education	176 (43%)	206 (47%)
Higher general education	82 (20%)	109 (25%)
Pre-university education	88 (22%)	86 (18%)
Combined educational level	42 (10%)	29 (7%)
Special needs education	21 (5%)	13 (3%)
Urbanicity of living environment^*b*^		
Urban	255 (62%)	260 (58%)
Suburban	79 (19%)	88 (20%)
Rural	80 (19%)	99 (22%)
Parental psychopathology, score^*c*^	0.14 (0.02, 0.21)	0.18 (0.04, 0.25)
Broad adverse life events^*d*^	3.32 (2.00, 4.00)	3.39 (2.00, 5.00)
Physical adverse life events, number^*e*^		
Total	107 (26%)	100 (22%)
Age up to 3	7 (1.7%)	8 (1.8%)
Age 4 to 8	22 (5.3%)	32 (7.2%)
Age 9 to 12	62 (15%)	28 (6.3%)
Age older than 12	29 (7.0%)	41 (9.2%)

^*a*^Mean (Interquartile range); n (%)

^*b*^Defined as number of addresses per km^2^ surrounding home address

^*c*^Measured by the Basic Symptom Inventory

^*d*^Sum count of the following categories: hospitalization of the adolescent; serious illness or hospitalization of the mother, father, sibling or close friend; death in the family or outside of family; parental divorce; repeating class; switching school; extended living away from home; physical violence and sexual abuse. See Additional file 1: Table S3 for full distribution of events across age groups

^*e*^Sum count exclusively of physical violence and sexual abuse. Presented if either occurred in a given age group

### Broad adverse life events and psychopathology

**Fig. 1:**
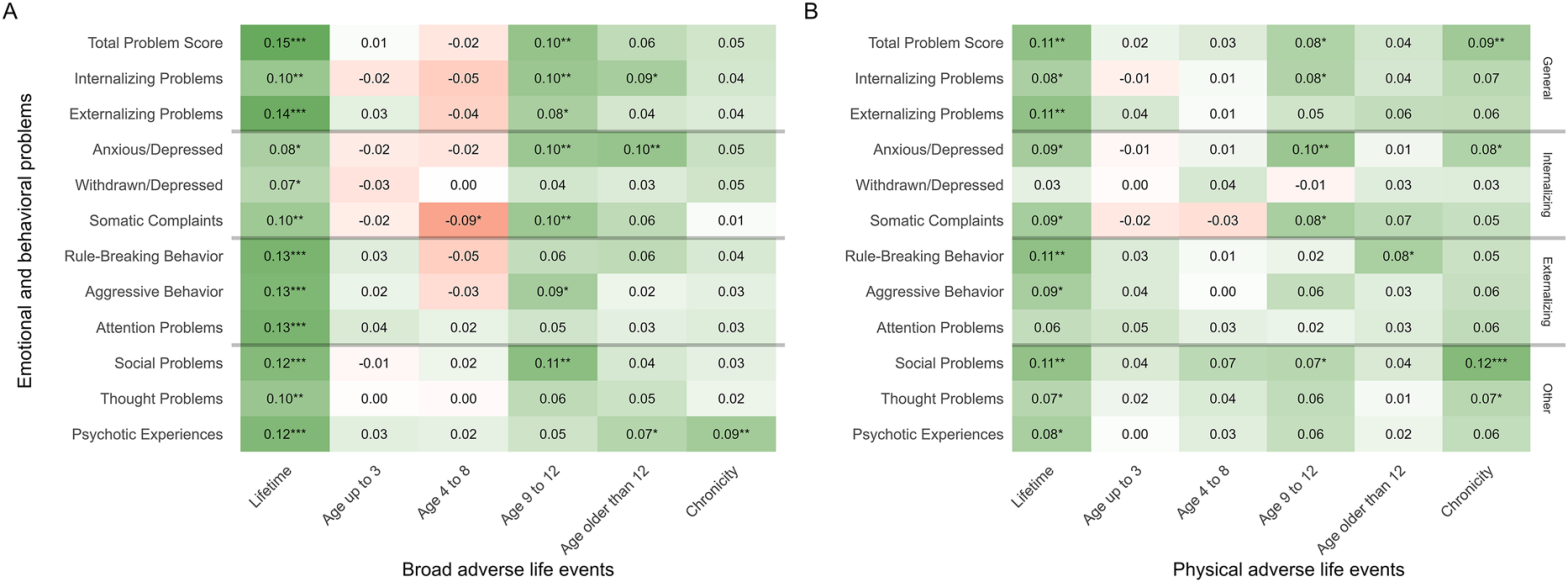
Standardized semi-partial r coefficients from associations between adverse life events (ALEs) and psychopathology outcomes. Results presented according to when ALEs occurred and their type. All estimates adjusted for sex, age, national origin, educational level, urbanization of living environment, harsh parenting of primary caregiver and parental psychopathology. Stars represent p-values, * p < 0.05, ** p < 0.01, *** p < 0.001. Broad ALEs consist of hospitalization of the adolescent; serious illness or hospitalization of the mother, father, sibling or close friend; death in the family or outside of family; parental divorce; repeating class; switching school; extended living away from home; physical violence and sexual abuse. Physical ALEs consist of physical violence and sexual abuse only

The adjusted semi-partial correlation coefficients between the broad operationalization of adverse life events and all psychopathology outcomes are visualized in Fig. 1A. Lifetime reporting of adverse life events were positively associated with all emotional and behavioral problems (partial r range 0.07–0.15). The largest partial correlations observed were with the total problem score (partial r = 0.15 [0.08, 0.21], p < 0.001), followed by externalizing problems (0.14 [0.08, 0.21], p < 0.001). The smallest associations were with internalizing problems such as the withdrawn/depressed (0.07 [0.00, 0.15], p = 0.043) and the anxious/depressed subscales (0.08 [0.01, 0.15], p = 0.017). Considering next the timing of broad adversity, we did not find associations in the early periods of life before age 3 or between age 4 and 8. The only exception was the only significant negative association with somatic complaints in ages 4–8 (− 0.09 [− 0.15, − 0.02], p = 0.014). Next, the period 9–12 years old was when most associations of statistical significance emerged, namely with total problems, externalizing, internalizing, anxious/depressed, somatic complaints, aggressive behavior and social problems scores (partial r range 0.09–0.11). There were no associations with scores reflecting attention problems, thought problems and psychotic experiences (partial r range 0.4–0.6). When looking at events happening after the age of 12, only psychotic experiences (0.07 [0.00, 0.14], p = 0.036) and internalizing problems (0.09 [0.02, 0.15], p = 0.013), in particular anxious/depressed problems (0.10 [0.03, 0.17], p = 0.003) were significantly associated. Chronicity of broad ALEs was not associated with any of the outcomes, except for psychotic experiences (0.09 [0.02, 0.16], p = 0.009). Full standardized semi-partial correlations are presented in Additional file 1: Table S5. Robustness to unmeasured confounding was strongest for the lifetime predictors, particularly the externalizing total problem score, rule-breaking behavior and psychotic experiences, all of which required a confounder 4 times bigger than parental psychopathology to remove the association. The externalizing problems subscales and psychotic experiences score were also fairly robust to confounding in the 9–12 age period, requiring a 3 times bigger confounder than parental psychopathology. All unmeasured confounding estimates are presented in Additional file 1: Fig. S1A.

### Physically threatening adverse life events and psychopathology

For the physical ALE predictors consisting of physical and sexual abuse, we present the adjusted semi-partial correlations with all psychopathology outcomes in Fig. 1B. As before, the most significant associations were found with the lifetime physical ALE count, however smaller positive correlations were observed (ranging from 0.11 to 0.03). In this analysis, there were also non-significant associations with the withdrawn/depressed score and attention problems. Again, we found no evidence of associations with early life physical ALEs in either the up to 3 or the 4 to 8 age period. The period between ages 9 to 12 had the highest number of significant associations. Those consisted of the anxious/depressed score (0.10 [0.03, 0.16], p = 0.005), somatic complaints (0.08 [0.02, 0.15], p = 0.016), total problems (0.08 [0.01, 0.14], p = 0.025), internalizing problems (0.08 [0.01, 0.14], p = 0.029) and social problems (0.07 [0.00, 0.14], p = 0.044). There were no significant associations in the most recent age group (older than 12), except for rule-breaking behavior (0.08 [0.02, 0.15], p = 0.015). Finally, more associations and some of the biggest effect sizes were found with chronicity of physical ALEs. Those were with social problems (0.12 [0.05, 0.19], p = 0.001), total problems (0.09 [0.02, 0.16], p = 0.008), anxious/depressed score (0.08 [0.01, 0.14], p = 0.029) and thought problems (0.07 [0.00, 0.14], p = 0.046). All associations with physical ALEs were considerably more robust to unmeasured confounding. Except the withdrawn/depressed outcome, all lifetime associations required an unmeasured confounder at least 3 times bigger than parental psychopathology to remove them. All associations in the 9 to 12 age period required a 5 times or bigger confounder to become zero. All unmeasured confounding estimates are presented in Additional file 1: Fig. S1B.

### The moderating effect of sex

**Fig. 2:**
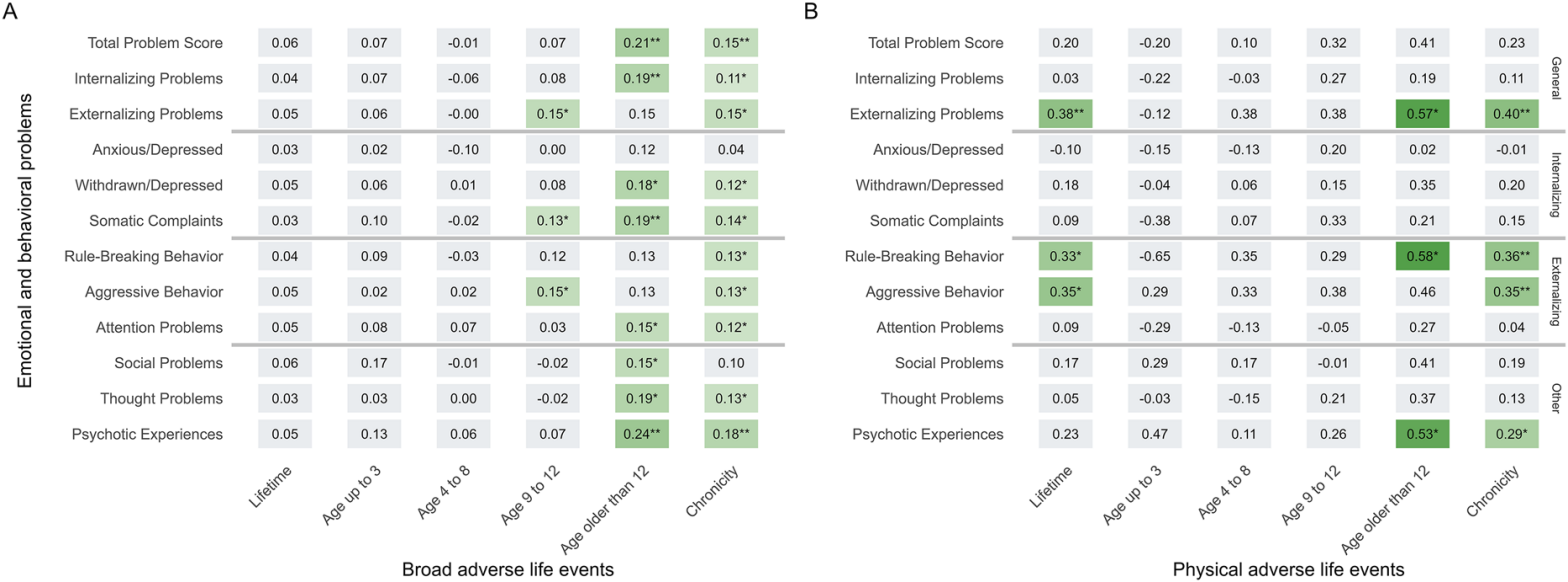
Standardized coefficients from interaction terms between sex and adverse life event (ALE) predictors. Results presented according to when ALEs occurred and their type. Significant moderation at p < 0.05 displayed in color. All estimates adjusted for sex, age, national origin, educational level, urbanization of living environment, harsh parenting of primary caregiver and parental psychopathology. Stars represent p-values, * p < 0.05, ** p < 0.01, *** p < 0.001. Broad ALEs consist of hospitalization of the adolescent; serious illness or hospitalization of the mother, father, sibling or close friend; death in the family or outside of family; parental divorce; repeating class; switching school; extended living away from home; physical violence and sexual abuse. Physical ALEs consist of physical violence and sexual abuse only

Finally, the standardized interaction coefficients of the analyses where a significant sex moderation was identified are presented in Fig. 2. All significant interaction terms shared a consistent feature—the positive standardized coefficients indicated the association between ALEs and all of the psychopathology outcomes were stronger among female adolescents than males. Thus, in all significant sex interaction models ALEs were a risk factor for psychopathology for female and not male adolescents. These sex differences are illustrated through estimated marginal means in Additional file 1: Fig. S2. Starting with Fig. 2A, there were no interactions identified for the lifetime broad ALEs predictors, signifying those association effect sizes were statistically indistinguishable between boys and girls. There were no significant interaction terms for the up to 3 years window, nor for the 4–8 years group. In the 9–12 period, there were interactions identified for externalizing problems (0.15 [0.02, 0.28], p = 0.020), in particular aggressive behavior (0.15 [0.02, 0.28], p = 0.024); indicating the associations were of bigger effect size among girls. A smaller interaction effect was also found for somatic complaints (0.13 [0.00, 0.25], p = 0.050). Turning to the older than 12 period, there were many interaction effects, which were previously not significant in their first order associations. The biggest effect sizes were also found in this window, psychotic experiences being the largest interaction (0.24 [0.09, 0.39], p = 0.001), followed by total problems (0.21 [0.07, 0.35], p = 0.004), internalizing problems (0.19 [0.05, 0.33], p = 0.009), withdrawn/depressed score (0.18 [0.04, 0.33], p = 0.013), somatic complaints (0.19 [0.05, 0.33], p = 0.007), thought problems (0.19 [0.04, 0.34], p = 0.014) and finally social problems (0.15 [0.01, 0.30], p = 0.040). Chronicity of broad ALEs was also moderated by sex across all psychopathology outcomes, with the exception of the anxious/depressed score and social problems outcomes. The standardized interaction coefficients ranged from 0.18 ([0.07, 0.29], p = 0.002) for psychotic experiences to 0.11 ([0.00, 0.21], p = 0.048) for internalizing problems. Interaction coefficients from all analyses are presented in Additional file 1: Table S6.

Turning to Fig. 2B, there were sex differences in the association between physical ALEs and psychopathology for 4 outcomes, 3 of which were related to externalizing behaviors. The first was the externalizing problems score, for which an interaction-effect was found for the lifetime physical ALE predictor (0.38 [0.11, 0.66], p = 0.007), the older than 12 age period (0.57 [0.08, 1.06], p = 0.022) and the chronicity operationalization (0.40 [0.13, 0.66], p = 0.003). Next, rule-breaking behavior was also moderated in its association with lifetime physical ALEs (0.33 [0.05, 0.60], p = 0.019), older than 12 age period (0.58 [0.10, 1.06], p = 0.019) and chronicity predictor (0.36 [0.10, 0.62], p = 0.007). The aggressive behavior association was moderated with sex for the lifetime physical ALEs (0.35 [0.07, 0.63], p = 0.013) and chronicity predictor (0.35 [0.08, 0.62], p = 0.010). Finally, for psychotic experiences the association between physical ALEs after age 12 (0.53 [0.04, 1.03], p = 0.034) and chronic physical ALEs (0.29 [0.03, 0.56], p = 0.032) was also moderated by sex. The effect sizes were relatively biggest for the older than 12 age period predictors, standardized interaction terms ranging from 0.53 to 0.58.

## Discussion

The current study examined three dimensions of ALEs (type, timing and sex differences) in their association with psychopathology outcomes. Several clear patterns emerged. First, the lifetime associations with broad ALEs (which included hospitalization, parental divorce, repeating a class) showed a bigger effect size than the physically threatening operationalization (which only included sexual and physical abuse). However, the latter category of ALEs was found to be more robust to unmeasured confounding and thus associations were less likely to arise due to an unobserved variable. Second, adverse events in later years, particularly between ages 9 to 12, were found to be most associated with psychopathology regardless of type of ALE. Due to low numbers of physical ALEs in the age period up to 3 years old, we could not provide strong evidence for associations in this earliest period. Third, a markedly consistent pattern of sex moderation emerged. Whenever there were sex differences in effects, girls were always estimated to have a stronger association between ALEs and psychopathology than boys. These differences were most often found in the broad operationalization of ALEs, particularly for events happening after age of 12 and for chronic ALEs. The implications of each of these general patterns are discussed.

### Differences between broad and physical adverse life events associations

Adolescents with more ALE in their lifetime history also self-reported more emotional, behavioral problems on all psychopathology outcomes measured. This positive lifetime association is solidly rooted in previous literature, which has reported similar links to internalizing/externalizing problems in children [21], young adults [68], and in later adulthood [42]. As in the current study, these associations remain after adjustment for socioeconomic factors and are robust when using official records to measure abuse [40, 49]. Going beyond previous literature, we found that using a broad operationalization of adversity produced bigger effect sizes on the associations with psychopathology than when limiting the definition only to physical and/or sexual adversity. This finding is perhaps not counterintuitive, considering that broad ALE predictor by definition contains more information about family dynamics, household dysfunction and the general environment growing up [8]. The results from the current study show this information can be exploited to more effectively predict mental health symptoms of any type. This better predictive value of broad ALEs, however, comes at a cost. Although the effect sizes were bigger for broad ALEs, the associations were also vulnerable to confounding by unmeasured factors. This suggests that the same environmental factors (e.g. household dysfunction), which are closely linked to broad ALEs, can both increase its predictive power and simultaneously confound its associations [79]. This has considerable implications for any theoretical model that attempts to causally map the effects of adversity. The physical ALE predictor, on the other hand, was found to have an association of smaller effect sizes with the psychopathology outcomes, but this time very large unmeasured confounders were needed to remove those associations. The current study therefore provides stronger evidence for physical and sexual abuse rather than broad ALEs as probable etiological contributors to psychopathology. The limited number of studies attempting to establish causal effects of adversity indeed support physical and sexual abuse as a causal factor for, for example, antisocial behavior and maladjustment [41, 76]. Taken together, this information points to a fundamental trade-off when using broad versus physically threatening operationalizations of ALEs. Broad ALEs provide better predictive value above and beyond key sociodemographic and parental factors. Physical ALEs are more suited for an etiological study into the mechanism behind psychopathology.

In terms of timing, we found the developmental period in the ages 9–12 was overall most often associated with psychopathology outcomes. This corroborates some empirical findings reported so far in the literature on adolescent mental health. For instance, a study which looked into harsh physical parenting and its timing effects on psychopathology found that age 9 was a particularly sensitive period [17]. Girls were most likely to develop internalizing problems at that age following harsh physical discipline, however boys were more sensitive at age 5. Another large cohort study focusing on children reports that middle childhood (around age 7) was when sexual and physical abuse produced most total psychopathology [21]. Physically assaultive events were also more predictive of later depression for events experienced in childhood (age < 12) than adolescence or adulthood in a twin study design [53]. There are, therefore, studies using children, adolescent and adult samples that report results similar to the present ones. Some researchers have made sense of these findings by positing that the beginning of puberty is a particularly sensitive period for adverse events due to accelerated hormonal and neurodevelopmental changes [51]. Equally however, there are studies that do not detect sensitive age periods for psychopathology after interpersonal violence exposure [22, 35]. Machine learning methods have also produced equivocal findings that adversity at ages 5–14 is most predictive of psychiatric symptoms [65] and adversity around age 5 is most predictive of positive psychosis symptoms [64]. These wide differences in designs, predictors, outcomes and methodology underline the importance of using outcome-wide approaches similar to the current study. The current results suggest that both internalizing and externalizing problems are most associated with adversity in the 9–12 age period.

### Heightened vulnerability of girls to ALE

The current study found that girls were at higher risk for nearly all externalizing, internalizing and cognitive outcomes after exposure to broad chronic ALEs or ALEs after age 12. Additionally, girls were at higher risk for externalizing problems after chronic physical ALEs (e.g. physical and sexual abuse), and physical ALEs after age 12. Analyses for ALEs after age 12 were adjusted for prior chronicity. Therefore, most likely, different mechanisms underlie these two findings. There were less sex differences with smaller effect sizes for lifetime and middle-childhood ALEs. Chronic adversity has previously appeared to be associated with psychopathology only with very small effect sizes when not considering the role of sex [23]. Studies that explicitly model sex differences, however, consistently report bigger effect sizes in girls exposed to adversity for depression [36], psychosis [29], PTSD [37] and delinquent behavior [48]. To our knowledge, this is the first study to show similar sex differences for chronic adversity and psychopathology. Notably, prior studies that investigated the timing component of sex differences report findings that parallel the present results. Breslau and colleagues (2017) used large-scale national survey data to study when girls surpass boys in reporting depression. Similar to the current sample, they estimate that age 12 is when statistically distinguishable differences emerge and continue to increase until around age 17. The present results suggest different responses to ALEs could be one explanation for that divergence. In a similar manner to the current analysis, Harkness et al. [36] studied how age moderates the effect of various ALEs with depressive disorders. Unlike the present results, they found that ALEs after age 17 was when women were significantly more vulnerable to adversity than men. The authors themselves, however, note a major limitation in their adolescent group that they had 17 boys in total. With the current bigger sample, we were able to show that events experienced in adolescence after age 12 were more associated with psychopathology for girls. This sex difference again has been theoretically attributed to differences in pubertal hormonal changes and prescribed affiliative gender norms for girls [13]. Although the mechanisms behind the sex differences remain ground for future research, the current investigation advances the knowledge about which ALEs could be prioritized for future study. Namely, chronic and late childhood experiences instead lifetime cumulative scores could be of particular research interest.

### Strengths and limitations

A range of limitations should be noted about the present study. First, the chosen mode of outcome-wide analysis was strictly on the exploratory end of the spectrum. In practice this meant we could only interpret general trends in the data which are extremely unlikely to be Type I errors (e.g. sex interactions for all outcomes being of the same direction, across all outcomes association concentrating in the middle childhood age group). Therefore, a substantial limitation is that we could not confidently conclude anything about the specific associations between predictors and outcomes (e.g. definitively conclude physical abuse in ages 9–12 is positively associated with psychotic experiences). Those types of conclusions require rigorous adjustments for multiple hypothesis testing. Even limiting the scope of the investigation to a subsection of outcomes would require statistical power far beyond the reach of existing cohorts, especially for the high-risk adolescent population we report on here. We have argued, however, that the general conclusions offered here still meaningfully advance our understanding of how ALEs and psychopathology relate. An added strength is that the current high-risk sample is a particularly adequate population for this research question, as many studies focusing on childhood adverse experiences use cases from social services or other governmental referrals [44]. In order for governmental intervention to occur, those cases selectively represent extreme and visible abuse, which do not capture the full range of less harsh adverse experiences [16]. Conversely, using parent-reported events could miss important events in the later years of the child or abuse perpetrated by the parents themselves [56]. Misclassifying abused adolescents as not abused could have conceivably biased associations towards the null. The current sample offers an advantageous balance of both heightened risk of reporting adversity, while covering a broad spectrum of experiences. There were physically threatening events we could study across all age and sex groups, except the earliest infancy period up to age 3 where events were reported only rarely. Due to the sampling strategy, the current high-risk cohort is not representative of the general population and thus the distributions of adversity and psychopathology are markedly distinct. However, the associations reported here can generalize to the general population due the inclusion of adolescents not at high risk of psychopathology [62]. Limitations also emerge from the cross-sectional nature of the study. It is conceivable that some reciprocal relationship exists between current levels of psychopathology and experiences of academic hardship or even parental dynamics resulting in divorce. Such a case is harder to make, however, for physical and sexual abuse events. We also took into account a wide range of socioeconomic variables, parental style and history of psychopathology, and prior chronic experiences of adversity when reporting on each association. We also conducted sensitivity analyses for unmeasured variables, which provided information on which associations are robust and which are likely to disappear under inevitable residual confounding.

## Conclusions

The current study shows when adversity may be most predictive of emotional and behavioral problems, especially in the middle-childhood years as adolescents transition into puberty. Furthermore, increased attention is warranted towards adolescent girls with chronic or recent experiences of broad adversity. Although physical or sexual abuse are striking events in an adolescent’s history, a girl with less conspicuous adversities might still be considered at a higher risk for developing emotional or behavioral problems. The operationalization of ALE as physically threatening, on the other hand, may be more useful in understanding etiological origins of psychopathology. As such, they are prime intervention targets to prevent future psychopathology of any kind. Future confirmatory studies could focus on any specific combination of ALEs and a psychopathology outcome to investigate other moderating mechanisms, changes over time and effectiveness of interventions like resilience training. Additionally, studies could investigate which protective factors attenuate the association with psychopathology in vulnerable age periods of childhood. A strong social support network or finding meaning in one’s experiences, for example, are some of the known factors that can lower or even reverse the effects of trauma into what is sometimes referred to as posttraumatic growth [57, 59]. Finally, we make recommendations on how ALE should be operationalized in practice. A nuanced decision should be made depending on the purpose of measuring ALEs. For prediction purposes, a broad operationalization of ALEs, including family divorce, school difficulties, and parental health, is best suited, even if the adolescent socioeconomic status and their parents’ mental health is known. This could be particularly useful for screening purposes, as there is already a strong movement in pediatric care to identify adversity early and promote practices for a supportive family environment [39]. Taken altogether, the current study provides information for which operationalizations of adversity are most associated with psychopathology, at what time point and in what sex.

### Supplementary Information

**Additional file1: Table S1.** Psychometric properties of psychopathology problem scales in the current sample. **Table S2.** Non-response analysis between participants with missing ALE or YSR data and those with complete data on those variables. **Table S3.** Adverse life events (ALEs) counts according to the age period in which they occurred. **Table S4.** Comparison of Internalizing, externalizing problems scores and psychotic experiences between boys and girls. **Table S5.** Standardized semi-partial r coefficients from associations between ALEs and psychopathology outcomes presented according to the time period in which ALEs occurred and their type. **Table S6.** Standardized beta coefficients from interaction terms between ALEs and sex with psychopathology scores as outcome. Results presented according to when ALEs occurred and their type. ﻿**Figure S1.** Robustness to unmeasured confounding for observed associations between adverse life events and the psychopathology outcomes. The numbers correspond to how many times bigger a simulated unmeasured confounder has to be than a measured confounder (we used parental psychopathology) to remove the association of interest. A value of 1x indicates unmeasured confounder as big as parental psychopathology can remove the association, whereas 5x+ corresponds to 5 times or bigger. **Figure S2.** Estimated marginal means probing the moderating effect of sex on ALE associations.

## Data Availability

All analysis files are publicly available at https://osf.io/8w7bp/. The data for the current study is not publicly available due to the sensitive nature of the clinical data.
