# Supplementary material for: Childhood adversity and psychopathology: the dimensions of timing, type and chronicity in a population-based sample of high-risk adolescents
Source: Child Adolesc Psychiatry Ment Health. 2024 Mar 18;18:37. doi: 10.1186/s13034-024-00727-x (PMC10949567; doi:10.1186/s13034-024-00727-x)
Supplement: Supplementary file 1 — Additional file1: Table S1. Psychometric properties of psychopathology problem scales in the current sample. Table S2. Non-response analysis between participants with missing ALE or YSR data and those with complete data on those variables. Table S3. Adverse life events (ALEs) counts according to the age period in which they occurred. Table S4. Comparison of Internalizing, externalizing problems scores and psychotic experiences between boys and girls. Table S5. Standardized semi-partial r coefficients from associations between ALEs and psychopathology outcomes presented according to the time period in which ALEs occurred and their type. Table S6. Standardized beta coefficients from interaction terms between ALEs and sex with psychopathology scores as outcome. Results presented according to when ALEs occurred and their type. ﻿Figure S1. Robustness to unmeasured confounding for observed associations between adverse life events and the psychopathology outcomes. The numbers correspond to how many times bigger a simulated unmeasured confounder has to be than a measured confounder (we used parental psychopathology) to remove the association of interest. A value of 1x indicates unmeasured confounder as big as parental psychopathology can remove the association, whereas 5x+ corresponds to 5 times or bigger. Figure S2. Estimated marginal means probing the moderating effect of sex on ALE associations. [file 13034_2024_727_MOESM1_ESM.docx]

Additional file materials

Childhood adversity and psychopathology: the dimensions of timing, type and chronicity in a population-based sample of high-risk adolescents

| Table S1. Psychometric properties of psychopathology problem scales in the current sample. | | | | | |
| --- | --- | --- | --- | --- | --- |
|  | **Items (n)** | Males | | Females | |
|  |  | **Cronbach's α** | **McDonald's ω** | **Cronbach's α** | **McDonald's ω** |
| **General problems** |  |  |  |  |  |
| Total problems | 105 | 0.92 [0.90, 0.93] | 0.92 | 0.95 [0.94, 0.95] | 0.95 |
| Internalizing problems | 31 | 0.86 [0.83, 0.88] | 0.88 | 0.90 [0.88, 0.91] | 0.91 |
| Externalizing problems | 32 | 0.82 [0.78, 0.84] | 0.84 | 0.86 [0.83, 0.88] | 0.89 |
| **Internalizing problems** | | |  |  |  |
| Anxious/Depressed | 13 | 0.81 [0.77, 0.84] | 0.84 | 0.86 [0.84, 0.88] | 0.89 |
| Withdrawn/Depressed | 8 | 0.68 [0.61, 0.73] | 0.74 | 0.73 [0.68, 0.77] | 0.79 |
| Somatic complaints | 10 | 0.72 [0.67, 0.76] | 0.77 | 0.74 [0.71, 0.78] | 0.80 |
| **Externalizing problems** | | |  |  |  |
| Rule-breaking | 15 | 0.66 [0.60, 0.71] | 0.72 | 0.71 [0.66, 0.75] | 0.77 |
| Aggressive behavior | 17 | 0.75 [0.72, 0.79] | 0.79 | 0.82 [0.78, 0.85] | 0.85 |
| Social problems | 11 | 0.53 [0.44, 0.60] | 0.63 | 0.66 [0.60, 0.70] | 0.73 |
| **Other problems** |  |  |  |  |  |
| Attention problems | 9 | 0.74 [0.70, 0.77] | 0.78 | 0.76 [0.72, 0.79] | 0.82 |
| Thought problems | 12 | 0.72 [0.66, 0.76] | 0.78 | 0.78 [0.74, 0.82] | 0.83 |
| Psychotic experiences | 16 | 0.74 [0.69, 0.78] | 0.79 | 0.79 [0.75, 0.81] | 0.82 |

| Table S2. Non-response analysis between participants with missing ALE or YSR data and those with complete data on those variables | | | |
| --- | --- | --- | --- |
|  | **Respondents**  N = 861*^1^* | **Non-respondents***^2^*  N = 161*^1^* | **p-value***^3^* |
| **Sex** |  |  | 0.215 |
| Male | 414 (48%) | 86 (53%) |  |
| Female | 447 (52%) | 75 (47%) |  |
| **Age, years** | 14.9 (14.4, 15.3) | 15.9 (14.9, 16.6) | <0.001 |
| **Ethnic origin** |  |  | 0.331 |
| Dutch | 646 (78%) | 63 (73%) |  |
| Non-Dutch | 184 (22%) | 23 (27%) |  |
| **Net monthly household income, euro’s** | | | 0.752 |
| < 1599€ | 98 (12%) | 10 (14%) |  |
| 1600 - 2399€ | 129 (16%) | 9 (13%) |  |
| 2400 – 4399€ | 401 (50%) | 33 (47%) |  |
| >4400€ | 175 (22%) | 18 (26%) |  |
| **Urbanicity of living environment** | | | 0.339 |
| Urban | 515 (60%) | 106 (66%) |  |
| Suburban | 167 (19%) | 25 (16%) |  |
| Rural | 179 (21%) | 30 (19%) |  |
| **Parental psychopathology, score** | 0.16 (0.04, 0.23) | 0.16 (0.04, 0.19) | 0.930 |
| **Educational level** |  |  | 0.013 |
| Pre-vocational education | 382 (45%) | 49 (46%) |  |
| Higher general education | 191 (22%) | 28 (26%) |  |
| Pre-university education | 174 (20%) | 11 (10%) |  |
| Combined education level | 71 (8.3%) | 17 (16%) |  |
| Special needs education | 34 (4.0%) | 2 (1.9%) |  |
| **Adolescent psychopathology problems, score** |  |  |  |
| Total problem score | 43.0 (28.0, 53.0) | 42.0 (28.0, 55.0) | 0.941 |
| Psychotic experiences | 3.3 (1.0, 5.0) | 3.2 (1.0, 5.0) | 0.746 |
| *^1^* n (%); Mean(IQR)  *^2^* Missing covariate counts in this group are 75 for national origin, 91 for household income, 136 for parental psychopathology, 54 for education and 53 for adolescent psychopathology | | | |
| *^3^*Pearson's Chi-squared test; Wilcoxon rank sum test | | | |

| Table S3. Adverse life events (ALEs) counts according to the age period in which they occurred | | | | |
| --- | --- | --- | --- | --- |
|  | Timing of ALE exposure | | | |
| **Adverse life event***^1^* | **Up to 3 years** | **4 to 8 years** | **9 to 12 years** | **Older than 12 years** |
| **Broad ALEs***^2^* |  |  |  |  |
| Hospitalization of adolescent | 241 (44.1%) | 86 (10.9%) | 56 (5.9%) | 29 (3.8%) |
| Serious illness or hospitalization of mother | 80 (14.6%) | 131 (16.6%) | 147 (15.5%) | 101 (13.3%) |
| Serious illness or hospitalization of father | 34 (6.2%) | 73 (9.2%) | 88 (9.3%) | 58 (7.7%) |
| Serious illness of sibling | 33 (6.0%) | 48 (6.1%) | 42 (4.4%) | 26 (3.4%) |
| Serious illness of friend | — | 1 (0.1%) | 12 (1.3%) | 10 (1.3%) |
| Death in the family | 4 (0.7%) | 10 (1.3%) | 6 (0.6%) | 5 (0.7%) |
| Death outside the family | — | 1 (0.1%) | 3 (0.3%) | 3 (0.4%) |
| Parental divorce | 65 (11.9%) | 97 (12.3%) | 80 (8.4%) | 43 (5.7%) |
| Repeated class | — | 7 (0.9%) | 126 (13.3%) | 144 (19.0%) |
| Switched schools | — | 82 (10.4%) | 101 (10.6%) | 99 (13.1%) |
| Extended living away from home | 8 (1.5%) | 6 (0.8%) | 5 (0.5%) | 4 (0.5%) |
| **Physically threatening ALEs***^3^* |  |  |  |  |
| Physical violence | 15 (2.7%) | 46 (5.8%) | 86 (9.1%) | 62 (8.2%) |
| Sexual abuse | 3 (0.5%) | 14 (1.8%) | 9 (0.9%) | 23 (3.0%) |
| *^1^* Percentages correspond to proportion a given life event makes up of all events in a given period. For example, parental divorce accounted for 11.9% of all events in the age up to 3 period.  *^2^* Broad ALE category includes all events, including physical violence and sexual abuse.  *^3^* Physically threatening ALE category consists of exclusively physical violence and sexual abuse | | | | |

| Table S4. Comparison of Internalizing, externalizing problems scores and psychotic experiences between boys and girls | | | |
| --- | --- | --- | --- |
|  | **Male**  N = 414*^1^* | **Female**  N = 447*^1^* | **p-value***^2^* |
| **General problems** |  |  |  |
| Total problems | 39.0 (26.4, 48.0) | 45.8 (30.0, 57.3) | <0.001 |
| Internalizing problems | 9.5 (4.0, 13.0) | 14.9 (8.3, 19.0) | <0.001 |
| Externalizing problems | 9.8 (5.0, 13.0) | 9.7 (5.0, 13.0) | 0.470 |
| **Internalizing problems** |  |  |  |
| Anxious/Depressed problems | 3.5 (1.0, 5.0) | 6.0 (2.0, 8.0) | <0.001 |
| Withdrawn/Depressed problems | 3.3 (1.0, 5.0) | 4.1 (2.0, 6.0) | <0.001 |
| Somatic problems | 2.7 (0.5, 4.0) | 4.8 (2.0, 7.0) | <0.001 |
| **Externalizing problems** |  |  |  |
| Rule-Breaking Behavior | 4.6 (2.6, 6.0) | 4.1 (2.0, 6.0) | 0.006 |
| Aggressive Behavior | 5.2 (2.0, 7.0) | 5.6 (2.0, 8.0) | 0.418 |
| Attention problems | 6.7 (4.0, 9.0) | 6.9 (5.0, 9.0) | 0.422 |
| **Other problems** |  |  |  |
| Social problems | 3.7 (2.0, 5.0) | 4.3 (2.0, 6.0) | 0.006 |
| Thought problems | 4.6 (2.0, 6.0) | 5.1 (2.0, 7.0) | 0.311 |
| Psychotic experiences | 3.2 (1.0, 4.3) | 3.5 (1.0, 5.0) | 0.365 |
| *^1^* Mean (Interquartile range) | | | |
| *^2^* Wilcoxon rank sum test | | | |

| Table S5. Standardized semi-partial r coefficients from associations between ALEs and psychopathology outcomes presented according to the time period in which ALEs occurred and their type | | | | | | |
| --- | --- | --- | --- | --- | --- | --- |
|  | Timing of ALE exposure | |  |  |  |  |
|  | **Lifetime** | **Up to 3 years** | **4 to 8 years** | **9 to 12 years** | **Older than 12 years** | **Chronic ALEs** |
| **Broad ALE predictors** | | | | | | |
| **General problems** |  |  |  |  |  |  |
| Total Problems | 0.15 [0.08, 0.21]*** | 0.01 [-0.06, 0.07] | -0.02 [-0.09, 0.05] | 0.10 [0.04, 0.17]** | 0.06 [-0.01, 0.13] | 0.05 [-0.02, 0.11] |
| Internalizing Problems | 0.10 [0.03, 0.17]** | -0.02 [-0.09, 0.04] | -0.05 [-0.11, 0.02] | 0.10 [0.03, 0.17]** | 0.09 [0.02, 0.15]* | 0.04 [-0.03, 0.11] |
| Externalizing Problems | 0.14 [0.08, 0.21]*** | 0.03 [-0.04, 0.09] | -0.04 [-0.11, 0.03] | 0.08 [0.02, 0.15]* | 0.04 [-0.03, 0.11] | 0.04 [-0.03, 0.10] |
| **Internalizing problems** | |  |  |  |  |  |
| Anxious/Depressed | 0.08 [0.01, 0.15]* | -0.02 [-0.09, 0.05] | -0.02 [-0.09, 0.04] | 0.10 [0.03, 0.16]** | 0.10 [0.03, 0.17]** | 0.05 [-0.02, 0.12] |
| Withdrawn/Depressed | 0.07 [0.00, 0.14]* | -0.03 [-0.09, 0.04] | 0.00 [-0.07, 0.07] | 0.04 [-0.02, 0.11] | 0.03 [-0.03, 0.10] | 0.05 [-0.02, 0.11] |
| Somatic Complaints | 0.10 [0.03, 0.16]** | -0.02 [-0.09, 0.05] | -0.09 [-0.15, -0.02]* | 0.10 [0.03, 0.16]** | 0.06 [-0.01, 0.13] | 0.01 [-0.06, 0.08] |
| **Externalizing problems** | |  |  |  |  |  |
| Rule-Breaking Behavior | 0.13 [0.06, 0.20]*** | 0.03 [-0.04, 0.10] | -0.05 [-0.11, 0.02] | 0.06 [-0.01, 0.13] | 0.06 [-0.00, 0.13] | 0.04 [-0.03, 0.11] |
| Aggressive Behavior | 0.13 [0.06, 0.19]*** | 0.02 [-0.05, 0.09] | -0.03 [-0.10, 0.04] | 0.09 [0.02, 0.15]* | 0.02 [-0.05, 0.09] | 0.03 [-0.04, 0.09] |
| Attention Problems | 0.13 [0.06, 0.19]*** | 0.04 [-0.03, 0.10] | 0.02 [-0.05, 0.09] | 0.05 [-0.02, 0.11] | 0.03 [-0.04, 0.09] | 0.03 [-0.04, 0.10] |
| **Other** **problems** |  |  |  |  |  |  |
| Social Problems | 0.12 [0.05, 0.19]*** | -0.01 [-0.08, 0.06] | 0.02 [-0.05, 0.08] | 0.11 [0.05, 0.18]** | 0.04 [-0.03, 0.11] | 0.03 [-0.04, 0.09] |
| Thought Problems | 0.10 [0.03, 0.17]** | -0.00 [-0.07, 0.07] | -0.00 [-0.07, 0.06] | 0.06 [-0.00, 0.13] | 0.05 [-0.02, 0.12] | 0.02 [-0.05, 0.09] |
| Psychotic experiences | 0.12 [0.05, 0.19]*** | 0.03 [-0.04, 0.10] | 0.02 [-0.04, 0.09] | 0.05 [-0.02, 0.12] | 0.07 [0.00, 0.14]* | 0.09 [0.02, 0.16]** |
| **Physical ALE predictors** | | | | | | |
| **General problems** |  |  |  |  |  |  |
| Total Problems | 0.11 [0.04, 0.18]** | 0.02 [-0.05, 0.09] | 0.03 [-0.04, 0.10] | 0.08 [0.01, 0.14]* | 0.04 [-0.02, 0.11] | 0.09 [0.02, 0.16]** |
| Internalizing problems | 0.08 [0.01, 0.15]* | -0.01 [-0.08, 0.06] | 0.01 [-0.06, 0.08] | 0.08 [0.01, 0.14]* | 0.04 [-0.03, 0.11] | 0.07 [-0.00, 0.14] |
| Externalizing problems | 0.11 [0.04, 0.17]** | 0.04 [-0.03, 0.11] | 0.01 [-0.06, 0.07] | 0.05 [-0.02, 0.11] | 0.06 [-0.01, 0.13] | 0.06 [-0.01, 0.13] |
| **Internalizing problems** | |  |  |  |  |  |
| Anxious/Depressed | 0.09 [0.02, 0.15]* | -0.01 [-0.08, 0.06] | 0.01 [-0.06, 0.08] | 0.10 [0.03, 0.16]** | 0.01 [-0.05, 0.08] | 0.08 [0.01, 0.14]* |
| Withdrawn/Depressed | 0.03 [-0.04, 0.09] | -0.00 [-0.07, 0.06] | 0.04 [-0.03, 0.11] | -0.01 [-0.08, 0.06] | 0.03 [-0.04, 0.10] | 0.03 [-0.04, 0.10] |
| Somatic Complaints | 0.09 [0.02, 0.15]* | -0.02 [-0.09, 0.05] | -0.03 [-0.10, 0.04] | 0.08 [0.02, 0.15]* | 0.07 [-0.00, 0.13] | 0.05 [-0.01, 0.12] |
| **Externalizing problems** | |  |  |  |  |  |
| Rule-Breaking Behavior | 0.11 [0.04, 0.18]** | 0.03 [-0.03, 0.10] | 0.01 [-0.06, 0.08] | 0.02 [-0.05, 0.09] | 0.08 [0.02, 0.15]* | 0.05 [-0.02, 0.12] |
| Aggressive Behavior | 0.09 [0.02, 0.15]* | 0.04 [-0.03, 0.10] | 0.00 [-0.06, 0.07] | 0.06 [-0.01, 0.12] | 0.03 [-0.04, 0.10] | 0.06 [-0.01, 0.12] |
| Attention Problems | 0.06 [-0.01, 0.13] | 0.05 [-0.02, 0.11] | 0.03 [-0.04, 0.09] | 0.02 [-0.05, 0.09] | 0.03 [-0.04, 0.10] | 0.06 [-0.01, 0.13] |
| **Other** **problems** |  |  |  |  |  |  |
| Social Problems | 0.11 [0.04, 0.17]** | 0.04 [-0.03, 0.10] | 0.07 [-0.00, 0.13] | 0.07 [0.00, 0.14]* | 0.04 [-0.03, 0.10] | 0.12 [0.05, 0.19]*** |
| Thought Problems | 0.07 [0.00, 0.14]* | 0.02 [-0.05, 0.08] | 0.04 [-0.03, 0.11] | 0.06 [-0.01, 0.13] | 0.01 [-0.06, 0.08] | 0.07 [0.00, 0.14]* |
| Psychotic experiences | 0.08 [0.02, 0.15]* | 0.00 [-0.06, 0.07] | 0.03 [-0.04, 0.10] | 0.06 [-0.01, 0.12] | 0.02 [-0.05, 0.09] | 0.06 [-0.01, 0.13] |
| All estimates adjusted for sex, age, national origin, educational level, urbanization of living environment, harsh parenting of primary caregiver and parental psychopathology. | | | | | | |


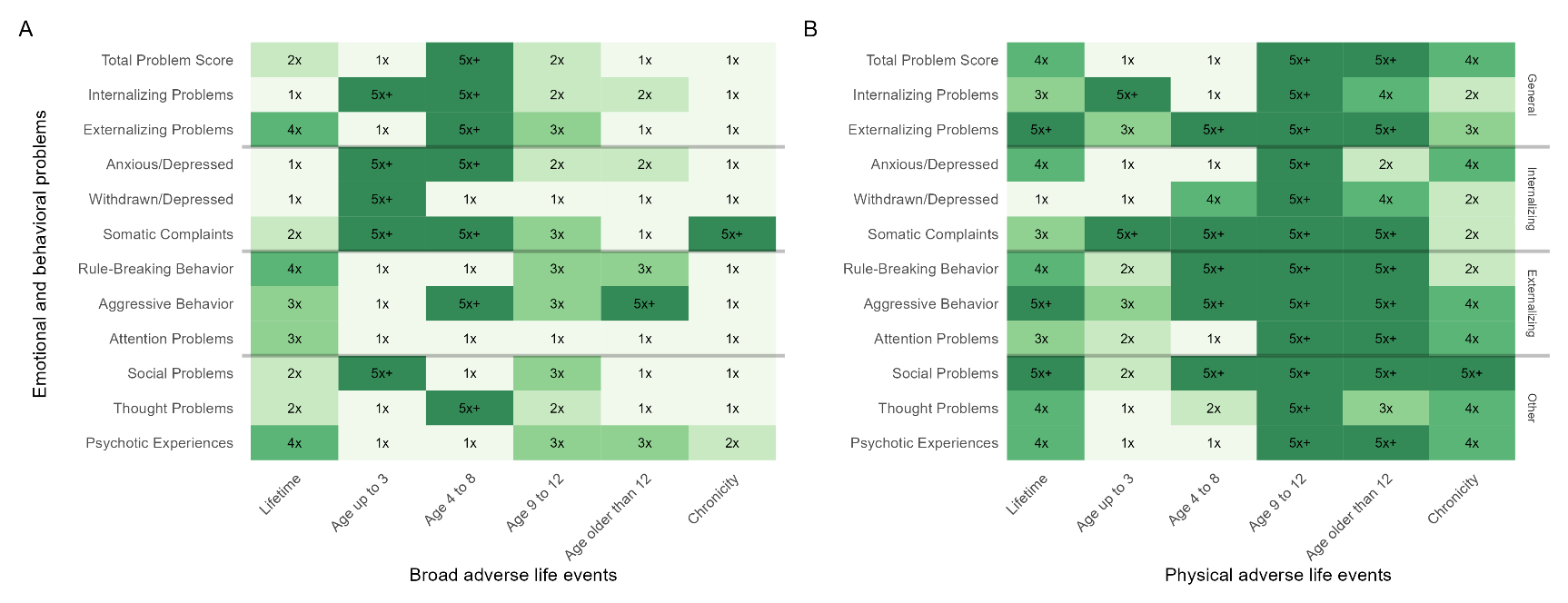
Figure S1. Robustness to unmeasured confounding for observed associations between adverse life events and the psychopathology outcomes. The numbers correspond to how many times bigger a simulated unmeasured confounder has to be than a measured confounder (we used parental psychopathology) to remove the association of interest. A value of 1x indicates unmeasured confounder as big as parental psychopathology can remove the association, whereas 5x+ corresponds to 5 times or bigger.

| Table S6. Standardized beta coefficients from interaction terms between ALEs and sex with psychopathology scores as outcome. Results presented according to when ALEs occurred and their type. | | | | | | |
| --- | --- | --- | --- | --- | --- | --- |
|  | Timing of ALE exposure | | **4 to 8 years** | **9 to 12 years** | **Older than 12 years** | **Chronic ALEs** |
|  | **Lifetime** | **Up to 3 years** |  |  |  |  |
| **Broad ALE predictors** | | | | | | |
| **General problems** |  |  |  |  |  |  |
| Total Problems | 0.06 [-0.01, 0.13] | 0.07 [-0.09, 0.23] | -0.01 [-0.14, 0.13] | 0.07 [-0.06, 0.20] | 0.21 [0.07, 0.35]** | 0.15 [0.04, 0.26]** |
| Internalizing Problems | 0.04 [-0.02, 0.11] | 0.07 [-0.09, 0.22] | -0.06 [-0.19, 0.07] | 0.08 [-0.04, 0.20] | 0.19 [0.05, 0.32]** | 0.11 [0.00, 0.21]* |
| Externalizing Problems | 0.05 [-0.02, 0.12] | 0.06 [-0.11, 0.23] | -0.00 [-0.14, 0.13] | 0.15 [0.02, 0.28]* | 0.15 [-0.00, 0.29] | 0.15 [0.03, 0.26]* |
| **Internalizing problems** | |  |  |  |  |  |
| Anxious/Depressed | 0.03 [-0.04, 0.10] | 0.02 [-0.14, 0.18] | -0.10 [-0.23, 0.03] | 0.00 [-0.12, 0.13] | 0.12 [-0.02, 0.26] | 0.04 [-0.07, 0.15] |
| Withdrawn/Depressed | 0.05 [-0.01, 0.12] | 0.06 [-0.10, 0.22] | 0.01 [-0.13, 0.14] | 0.08 [-0.05, 0.21] | 0.18 [0.04, 0.33]* | 0.12 [0.01, 0.23]* |
| Somatic Complaints | 0.03 [-0.04, 0.10] | 0.10 [-0.06, 0.26] | -0.02 [-0.15, 0.11] | 0.13 [0.00, 0.25]* | 0.19 [0.05, 0.33]** | 0.14 [0.03, 0.25]* |
| **Externalizing problems** | |  |  |  |  |  |
| Rule-Breaking Behavior | 0.04 [-0.03, 0.11] | 0.09 [-0.07, 0.26] | -0.03 [-0.17, 0.10] | 0.12 [-0.01, 0.25] | 0.13 [-0.02, 0.27] | 0.13 [0.02, 0.24]* |
| Aggressive Behavior | 0.05 [-0.02, 0.12] | 0.02 [-0.15, 0.19] | 0.02 [-0.12, 0.16] | 0.15 [0.02, 0.28]* | 0.13 [-0.01, 0.28] | 0.13 [0.02, 0.24]* |
| Attention Problems | 0.05 [-0.02, 0.12] | 0.08 [-0.09, 0.24] | 0.07 [-0.06, 0.21] | 0.03 [-0.10, 0.17] | 0.15 [0.00, 0.30]* | 0.12 [0.01, 0.24]* |
| **Other problems** |  |  |  |  |  |  |
| Social Problems | 0.06 [-0.01, 0.13] | 0.17 [-0.00, 0.33] | -0.01 [-0.14, 0.13] | -0.02 [-0.15, 0.11] | 0.15 [0.01, 0.30]* | 0.10 [-0.01, 0.22] |
| Thought Problems | 0.03 [-0.04, 0.10] | 0.03 [-0.14, 0.20] | 0.00 [-0.14, 0.14] | -0.02 [-0.15, 0.12] | 0.19 [0.04, 0.34]* | 0.13 [0.02, 0.25]* |
| Psychotic experiences | 0.05 [-0.02, 0.12] | 0.13 [-0.04, 0.29] | 0.06 [-0.08, 0.19] | 0.07 [-0.06, 0.20] | 0.24 [0.09, 0.39]** | 0.18 [0.07, 0.29]** |
| **Physical ALE predictors** | | | | | | |
| **General problems** |  |  |  |  |  |  |
| Total Problems | 0.20 [-0.08, 0.47] | -0.20 [-1.23, 0.83] | 0.10 [-0.44, 0.64] | 0.32 [-0.14, 0.77] | 0.41 [-0.08, 0.89] | 0.23 [-0.02, 0.49] |
| Internalizing Problems | 0.03 [-0.23, 0.30] | -0.22 [-1.27, 0.82] | -0.03 [-0.55, 0.49] | 0.27 [-0.17, 0.71] | 0.19 [-0.27, 0.66] | 0.11 [-0.14, 0.36] |
| Externalizing Problems | 0.38 [0.11, 0.66]** | -0.12 [-1.17, 0.92] | 0.38 [-0.17, 0.93] | 0.38 [-0.09, 0.84] | 0.57 [0.08, 1.06]* | 0.40 [0.13, 0.66]** |
| **Internalizing problems** | |  |  |  |  |  |
| Anxious/Depressed | -0.10 [-0.37, 0.17] | -0.15 [-1.17, 0.87] | -0.13 [-0.67, 0.40] | 0.20 [-0.25, 0.66] | 0.02 [-0.46, 0.50] | -0.01 [-0.26, 0.25] |
| Withdrawn/Depressed | 0.18 [-0.10, 0.45] | -0.04 [-1.07, 1.00] | 0.06 [-0.49, 0.61] | 0.15 [-0.32, 0.61] | 0.35 [-0.13, 0.84] | 0.20 [-0.06, 0.46] |
| Somatic Complaints | 0.09 [-0.17, 0.36] | -0.38 [-1.41, 0.66] | 0.07 [-0.45, 0.59] | 0.33 [-0.11, 0.78] | 0.21 [-0.25, 0.68] | 0.15 [-0.11, 0.40] |
| **Externalizing problems** | |  |  |  |  |  |
| Rule-Breaking Behavior | 0.33 [0.05, 0.60]* | -0.65 [-1.69, 0.40] | 0.35 [-0.20, 0.90] | 0.29 [-0.17, 0.75] | 0.58 [0.10, 1.06]* | 0.36 [0.10, 0.62]** |
| Aggressive Behavior | 0.35 [0.07, 0.63]* | 0.29 [-0.77, 1.34] | 0.33 [-0.23, 0.89] | 0.38 [-0.09, 0.85] | 0.46 [-0.04, 0.95] | 0.35 [0.08, 0.62]** |
| Attention Problems | 0.09 [-0.19, 0.37] | -0.29 [-1.35, 0.77] | -0.13 [-0.69, 0.43] | -0.05 [-0.52, 0.42] | 0.27 [-0.23, 0.76] | 0.04 [-0.23, 0.31] |
| **Other problems** |  |  |  |  |  |  |
| Social Problems | 0.17 [-0.11, 0.45] | 0.29 [-0.77, 1.35] | 0.17 [-0.39, 0.73] | -0.01 [-0.48, 0.46] | 0.41 [-0.08, 0.91] | 0.19 [-0.07, 0.46] |
| Thought Problems | 0.05 [-0.23, 0.34] | -0.03 [-1.10, 1.04] | -0.15 [-0.72, 0.41] | 0.21 [-0.27, 0.68] | 0.37 [-0.13, 0.87] | 0.13 [-0.14, 0.40] |
| Psychotic experiences | 0.23 [-0.05, 0.51] | 0.47 [-0.59, 1.52] | 0.11 [-0.44, 0.67] | 0.26 [-0.21, 0.73] | 0.53 [0.04, 1.03]* | 0.29 [0.03, 0.56]* |
| All estimates adjusted for sex, age, national origin, educational level, urbanization of living environment, harsh parenting of primary caregiver and parental psychopathology. | | | | | | |


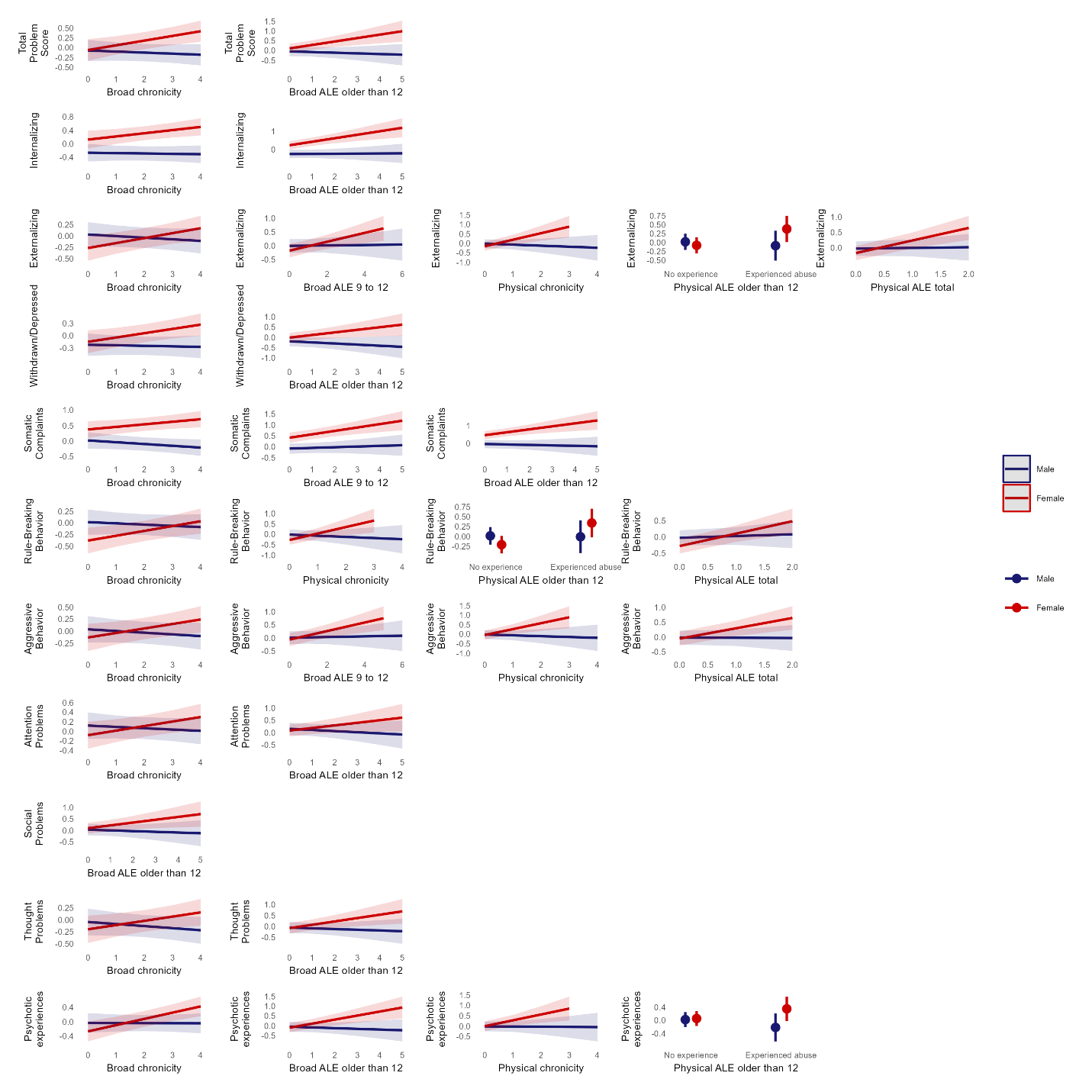
Figure S2. Estimated marginal means probing the moderating effect of sex on ALE associations.

Presented only the statistically significant interaction effects for purposes of conserving space. Notably all statistically significant interactions indicate a stronger associations for female adolescents than for males.
